# Sarcopenia and Treatment Toxicity in Older Adults Undergoing Chemoradiation for Head and Neck Cancer: Identifying Factors to Predict Frailty

**DOI:** 10.3390/cancers14092094

**Published:** 2022-04-22

**Authors:** Ryan T. Morse, Rohit G. Ganju, Gregory N. Gan, Ying Cao, Prakash Neupane, Kiran Kakarala, Yelizaveta Shnayder, Christopher E. Lominska

**Affiliations:** 1 Department of Radiation Oncology, UNC Lineberger Comprehensive Cancer Center, University of North Carolina Hospital, Chapel Hill, NC 27514, USA; 2 Department of Radiation Oncology, Wellstar Health System, Marietta, GA 30067, USA; guganju@gmail.com; 3 Department of Radiation Oncology, University of Kansas Cancer Center, Kansas City, KS 66160, USA; ggan@kumc.edu (G.N.G.); ycao@kumc.edu (Y.C.); clominska@kumc.edu (C.E.L.); 4 Department of Medical Oncology, University of Kansas Cancer Center, Kansas City, KS 66160, USA; pneupane@kumc.edu; 5 Department of Otolaryngology, University of Kansas Cancer Center, Kansas City, KS 66160, USA; kkakarala@kumc.edu (K.K.); yshnayder@kumc.edu (Y.S.)

**Keywords:** geriatric oncology, geriatric assessment, sarcopenia, treatment toxicity, chemotherapy

## Abstract

**Simple Summary:**

Objective frailty measures that predict treatment related toxicities for older adults are not well represented in the literature. In this study we identified pretreatment factors including sarcopenia, or loss of muscle mass, that may predict for patients experiencing increased treatment toxicity and requiring breaks from treatment. Based on the results of our study, sarcopenia may be used as an additional marker for frailty alongside traditional performance status scales. Prospective research is needed to identify subsets of older patients at risk for severe toxicity who might benefit from intensive supportive care to maximize oncologic outcomes while maintaining quality of life.

**Abstract:**

This study was performed to identify treatment related toxicities in older adults undergoing concurrent chemoradiotherapy for head and neck cancer and nutritional and skeletal muscle measures that might identify frailty. Imaging analysis was done with the following skeletal muscle measurements: skeletal muscle index (SMI), skeletal muscle density (SMD), and skeletal muscle gauge (SMG). Patients were dichotomized by age into younger (<70 years old, 221 patients) and older age groups (≥70 years old, 51 patients). Low SMI was more common in older patients (86.7%) compared to younger patients (51.7%, *p* < 0.01), as were low SMD (57.8% vs. 37.3%, *p* = 0.012) and low SMG (76.1% vs. 44.2%, *p* < 0.01), despite having similar BMIs (27.3 kg/m^2^ versus 27.7 kg/m^2^, *p* = 0.71). Older patients were significantly more likely to experience chemotherapy toxicity than younger patients (54.9% versus 32.3%, *p* < 0.01). On multivariate analysis age (*p* < 0.01), current smoking status (*p* < 0.01), and low SMI (*p* < 0.01) remained as significant predictors for missed chemotherapy cycles or discontinuation. Older patients were more likely to require ≥5-day radiation breaks than younger patients (27.5% versus 8.6%, *p* < 0.01). On multivariate analysis, age (*p* < 0.01), low albumin status (*p* = 0.03), and low SMI (*p* = 0.04) were identified as predictors of prolonged radiation treatment breaks. Based on the results of our study, sarcopenia may be used as an additional marker for frailty alongside traditional performance status scales.

## 1. Introduction

The geriatric population is a unique entity within the field of oncology and older adults make up a substantial proportion of patients diagnosed with cancer. In the United States, the incidence of patients diagnosed with cancer ≥ 65 years of age will soon reach approximately 70% [1]. A paucity of data exists to help guide management options in these older age individuals [2,3]. As oncology outcomes have continued to improve and de-escalation trials have shown promising results, little information can be applied to older populations, as elderly patients frequently are excluded or under-represented in cancer clinical trials, with a median enrollment age of 60 years [4]. Oncology organizations have recognized that standard oncology performance measures are inadequate for the assessment of older patients and that additional measures are needed to identify an individual based on physiologic age and not chronological age [2,5,6].

National organizations including ASCO and the U.S. FDA have developed strategies to address gaps in caring for older adults with cancer by developing education tools and guidelines for oncologists [7,8,9]. ASCO created their first formal guideline in 2018, recommending routine clinical use of geriatric tools to identify functional status, comorbidity, falls, depression, cognition, and nutrition [10]. Tools to assess vulnerability in geriatric patients have been shown to decrease treatment toxicity [11,12]; however, they are infrequently used in routine practice [7].

Patients with locally advanced head and neck cancer typically experience significant toxicity related to their cancer and from aggressive treatment, with the general treatment paradigm being concurrent chemotherapy and radiation [13]. Detecting pre-treatment factors that predispose patients to treatment toxicity could help clinicians make informed decisions regarding treatment choices or patients who may benefit from aggressive supportive care to limit treatment breaks. Frailty in older adults may be diagnosed by a comprehensive geriatric assessment, but these comprehensive assessments can be time-consuming. Recent evidence has shown that sarcopenia, defined as a loss of skeletal muscle mass, may be a biomarker for frailty in older adults with head and neck cancer, and that sarcopenia may serve as an alternative to commonly used geriatric screening tools identifying frail individuals [14]. The risk of sarcopenia has not particularly been explored in older populations with head and neck cancer relative to younger patients and how it affects treatment related toxicity. This study was performed to identify these treatment related toxicities and whether nutritional and skeletal muscle measures, particularly sarcopenia, may be additional markers of frailty.

## 2. Materials and Methods

### 2.1. Patient Selection

The medical records of consecutive patients with histologically proven head and neck cancer (HNC) at a single institution treated concurrently with chemoradiation (CRT) between 2011 and 2016 were retrospectively reviewed. Patients included all had Stage III, IVA, or IVB disease by the AJCC 7th edition; patients with Stage IVC were excluded [15]. Cutaneous squamous cell carcinomas and primary squamous cell cancers of occult origin metastatic to the cervical lymph node were excluded. All patients were presented at a multidisciplinary head and neck tumor conference for treatment recommendations. After obtaining institutional review board approval (IRB 1335), demographic, treatment, and outcomes data were extracted from the electronic medical records. Testing for high-risk human papilloma virus (HPV) status was obtained by immunochemical staining, using p16 as the surrogate marker. Serum values were collected prior to commencement of any chemotherapy and radiation. The neutrophil to lymphocyte ratio (NLR) ratio was calculated by dividing the absolute neutrophil count by the absolute lymphocyte count. Eastern Cooperative Oncology Group (ECOG) Performance Status was recorded to account for differences in baseline performance status [16]. Patients were dichotomized by the age at diagnosis into younger (<70 years old) and older age groups (≥70 years old).

### 2.2. Treatment and Toxicity

All patients in this study received intensity modulated radiation therapy (IMRT) with standard fractionation. Standard radiation dosing in the definitive setting was 70 Gray (Gy) and 60–66 Gy in the postoperative setting per established risk factors [17]. Radiation dose was not adjusted for low-risk patients, comorbidity, or elderly status. The choice of concurrent chemotherapy and induction chemotherapy, if applicable, was made at the discretion of the treating medical oncologist. Per institutional policy, patients deemed appropriate cisplatin candidates should receive cisplatin. Currently, institutional standards do not have specified cutoff values (i.e., ECOG performance status ≥2, creatinine clearance (CrCl) <60 mL/min) or require formal audiology testing to determine cisplatin ineligibility. Radiation toxicity was defined as any missed treatments due to toxicity; days missed due to holidays, machine maintenance, travel issues, or weekend days were not recorded as days missed. Toxicity for chemotherapy agents was defined as delays greater than 1 week in therapy administration or failure to complete all planned cycles of chemotherapy. Specific toxicities were classified by Common Terminology Criteria of Adverse Events (version 4.0). Only toxicities that occurred during concurrent chemotherapy administration, rather than induction chemotherapy, were included.

### 2.3. Body Composition Measurement

**Figure 1:**
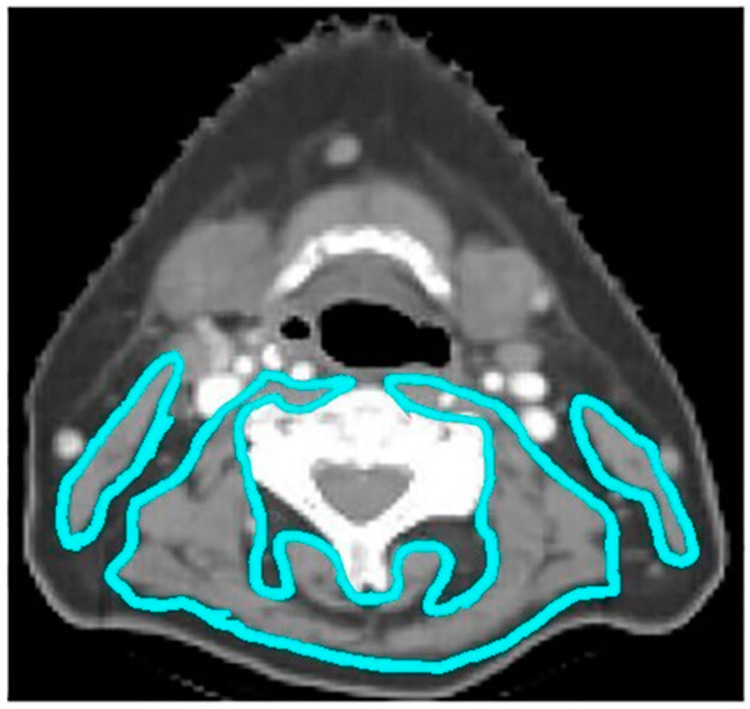
A representative case example of a 54-year-old man with an HPV-positive oropharynx cancer. A single axial CT slice is taken at the C3 vertebral body level with contours of the sternocleidomastoid and paravertebral muscles delineated in turquoise. Skeletal muscle was defined as −29 to +150 Hounsfield Units (HUs), and the total cross-sectional area (CSA) was computed automatically within the contoured perimeters.

Computed tomography (CT) scans were obtained for all patients at the time of radiation simulation for treatment planning purposes. Body Mass Index (BMI) was calculated using the patient’s weight in kilograms divided by their height in meters squared. Muscle composition was assessed using three different skeletal muscle measures: skeletal muscle index (SMI), skeletal muscle density (SMD), and skeletal muscle gauge (SMG). Skeletal muscle measurements were obtained from treatment planning CT scans using National Institutes of Health (NIH) ImageJ software. All skeletal muscle contouring was performed by a single researcher who was not aware of patient outcomes (R.G.G). An example from this dataset is shown in Figure 1.

The presence of sarcopenia was assessed using SMI, a validated method using CT-based measurements to calculate skeletal muscle mass [18,19]. SMI was calculated as previously described by Swartz et al. using a single axial CT slice at the C3 vertebral body level [19]. SMD was calculated using the mean attenuation within the same contoured perimeter. SMI and SMD thresholds were made consistent with thresholds that have been associated with increased mortality in a large cohort of cancer patients [20]. SMG was calculated as the product of SMI and SMD as described by Weinberg et al. [21]. Patients were dichotomized around the median in the overall cohort into low and high SMG groups for data analysis.

### 2.4. Statistical Analysis

Differences between groups were compared with a Student’s *t*-test for continuous variables and Pearson’s Chi-square or Fisher’s exact test, as appropriate, for categorical variables. Univariate (UVA) and multivariate analyses (MVA) were performed using logistic regression models to identify factors associated with chemotherapy and radiation treatment breaks. Significant variables (global *p*-value of <0.05) from the UVA and important variables identified by random forest method were included to fit the multivariable model [22]. Statistical analyses were conducted using Statistical Analysis System version 9.4 (SAS Institute, Cary, NC, USA). All tests were 2-sided and a *p*-value of 0.05 was considered significant.

## 3. Results

### 3.1. Patients

A total of 272 patients receiving CRT were identified with a median follow up of 34.3 months (range, 0.80–83.3). The population was mostly white (90.1%). The most common subsite was oropharynx (64.7%), 137 (77.8%) of which were p16-positive. All patients received CRT, including 22 patients receiving induction therapy. Patient characteristics by younger (<70, *n* = 221) and older (≥70, *n* = 51) age can be seen in Table 1. Older patients had expected higher ECOG performance status ≥2 pre-treatment (19.6% versus 8.6%, *p* = 0.022) compared to younger patients, however there was also a higher proportion of p16 positive oropharynx cancer patients in the older group (62.7% versus 47.5%, *p* = 0.050). Clinical and demographic characteristics were otherwise well balanced between the two groups (*p* > 0.05). Similar amounts of older and younger patients were treated with upfront surgical resection (25.5% vs. 19.9%, *p* = 0.37) and limited patients underwent induction chemotherapy before CRT (2.0% vs. 9.5%, *p* = 0.08).

Baseline nutritional serum values and skeletal muscle measurements were collected in each patient. An NLR ≥ 3 occurred in 152 (56.3%) patients and 32 (11.9%) patients met criteria for hypoalbuminemia (≤3.5 g/dL). NLR was significantly higher in older patients compared to younger patients (68.6% vs. 53.4%, *p* = 0.048). Before initiation of CRT, similar amounts of patients had hypoalbuminemia (13.7% older vs. 11.4% younger, *p* = 0.646). Skeletal muscle measurements consisted of SMI, SMD, and SMG with below threshold frequencies of 58.1%, 41.4%, and 50.0%, respectively, in the total cohort. Sarcopenia, determined by a low SMI, was more common in older patients (86.7%) compared to younger patients (51.7%, *p* < 0.01) despite having similar BMIs (27.3 kg/m^2^ vs. 27.7 kg/m^2^, *p* = 0.71). Low SMD (57.8% vs. 37.3%, *p* = 0.012) and low SMG (76.1% vs 44.2%, *p* < 0.01) were significantly more common in older patients compared to younger patients.

### 3.2. Treatment Toxicity

Ninety-nine patients (36.5%) experienced chemotherapy toxicity, defined as delays of one week or greater or were unable to complete their chemotherapy. On UVA, older patients were significantly more likely to experience chemotherapy toxicity than younger patients (54.9% versus 32.3%, *p* < 0.01) (Table S1). MVA was performed to identify factors associated with chemotherapy treatment breaks (Table 2); age ≥70 (*p* < 0.01), current smoking status (*p* < 0.01), and sarcopenia (*p* < 0.01) remained as significant predictors. Cisplatin chemotherapy was more commonly received by younger patients (62% high-dose triweekly dosing, 38% weekly dosing) compared to older patients (15% high-dose triweekly dosing, 85% weekly dosing) concurrent with radiation (74.7% vs. 52.9%, *p* < 0.01), as opposed to other cytotoxic chemotherapies. The mean total dose cisplatin received for younger patients was 241.7 mg/m^2^ and mean total dose for older patients was 192.1 mg/m^2^ (*p* < 0.01). The cisplatin patients receiving at least 200 mg/m^2^ in the younger cohort was 83.6% and in the older cohort was 63.0% (*p* = 0.012). Among patients receiving cisplatin, few patients experienced CTCAE acute kidney injury ≥ grade 2 (17.6% younger vs. 8.3% older patients, *p* = 0.38). Patients experiencing CTCAE neutropenia ≥ grade 2 were similar (44.8% younger versus 33.3% older patients, *p* = 0.30).

**Figure 2:**
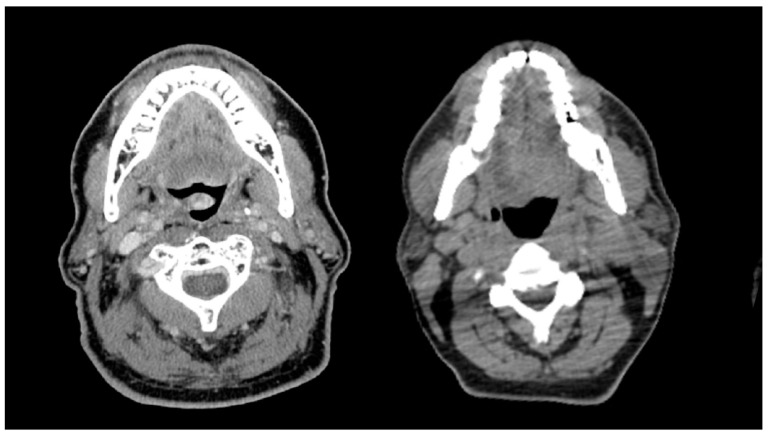
A comparison of older and younger patients with and without sarcopenia with similar BMI. The left panel represents a 70-year-old man with HPV-positive oropharynx cancer with sarcopenia (BMI 27.6 kg/m²) experiencing 10 days of radiation treatment delay, while the right panel represents a 56-year-old man with HPV-positive oropharynx cancer without sarcopenia (BMI 26.7 kg/m²) having no days of radiation treatment delay.

Thirty-four percent of patients experienced at least one missed radiation day due to toxicity, with a median of two treatments missed (range, 1–30). On UVA, Older patients required more radiation treatment breaks compared to younger patients (51.0% versus 29.9%, *p* < 0.01) (Table S1). MVA to identify factors associated with at least one radiation treatment break (Table 2) found only age ≥70 as a predictor (*p* = 0.012). Twelve percent of patients required a prolonged radiation treatment break, defined as a break of 1 week or greater. The most common reason for prolonged break was CTCAE grade 3 mucositis (36.3%). Older patients were more likely to require prolonged treatment breaks than younger patients on UVA (27.5% versus 8.6%, *p* < 0.01) (Table S1). On MVA (Table 2), age ≥70 (*p* < 0.01), low albumin status (*p* = 0.03), and sarcopenia (*p* = 0.04) were identified as predictors of prolonged radiation treatment breaks. Representative CT slices comparing radiation toxicity of an older adult with sarcopenia and younger adult without sarcopenia are shown in Figure 2. Nutritional dependence on PEG tube (CTCAE grade 3) was similar between groups (65.6% younger versus 66.7% older, *p* = 0.89). One-hundred six patients lost greater than 10% of their pretreatment body weight (42.5% younger versus 23.5% older patients, *p* = 0.012). Patients requiring hospital admission (CTCAE grade 3) during treatment occurred in 68 total patients (25.0%), with a trend toward older patients (35.3% versus 22.6%, *p* = 0.060).

## 4. Discussion

Predictive measures of treatment toxicity in older adults undergoing definitive treatment for head and neck cancer is not well represented in the literature. In this study we aimed to identify pretreatment factors, specifically sarcopenia, that may predict for patients experiencing increased treatment toxicity and requiring breaks from treatment. We studied how this may impact older adults compared to younger patients, who may have less physiologic reserve to withstand aggressive treatment. We found several factors associated with chemotherapy and radiation treatment toxicity, which typically were more prevalent in older adults. Sarcopenia was identified as a pretreatment characteristic predictive of chemotherapy toxicity and prolonged radiation break and was present in 86% of elderly patients. Based on the results of our study, sarcopenia may be used as an additional marker for frailty alongside traditional performance status scales.

Sarcopenia is thought to be a sensitive marker for the pro-inflammatory state of a patient’s cancer [23]. This marker is thought to be related to the wasting syndrome of cancer cachexia, although the mechanism is not fully known [20] and has been identified as an independent prognostic factor for treatment-related toxicity, overall survival, and progression-free survival within many solid tumors, including head and neck cancer [20,24,25,26]. Based on the results of our study, a potential mechanism could be related to frequent treatment breaks in radiation, which can worsen outcomes in HNC due to the accelerated repopulation of cancer cells and increased radioresistance [27]. Sarcopenia may be a clinically distinct “frailty syndrome” marked by declines in physiologic reserve and a resulting inability to manage acute stressors [28]. These patients may be less suited to tolerate the significant toxicities that accompany CRT in HNC. However, sarcopenia may be a modifiable risk factor. Improvements in nutritional and physical status before treatment initiation may counterbalance the pro-inflammatory state, leading to less treatment breaks and toxicities. Intense nutritional interventions in HNC patients undergoing CRT have been shown to minimize weight loss and improve treatment tolerance [29]. Results from the DAHANCA 25 trial demonstrated that progressive resistance training improved lean body mass and functional performance following radiotherapy and the DAHANCA 31 trial will be measuring outcomes for HNC patients during CRT [30,31].

Recommendations from ASCO to utilize geriatric assessments in older adults have led to increased use in medical oncology practice. In a phase III trial, Li et al. enrolled 605 older adults starting a new chemotherapy regimen to geriatric assessment-driven interventions (GAIN trial) and showed a 10.1% absolute reduction in grade 3 or higher chemotherapy-related toxic effects [32]. Mohile et al. enrolled patients 70 years and older starting a new chemotherapy treatment regimen to tailored recommendations based on impaired geriatric assessment domains (GAP70+ trial) and showed a 20% absolute reduction in grade 3–5 toxic effects, reduced falls, and reduced rates of polypharmacy [33]. Comprehensive geriatric assessments for patients starting chemotherapy have also provided prospective evidence leading to improvements in quality of life, reduced unplanned hospital admissions, and lower rates of treatment discontinuation [34]. Limited prospective data is available that specifically uses geriatric assessments in older adults undergoing definitive radiotherapy to evaluate for treatment related adverse events. VanderWalde et al. prospectively performed pretreatment comprehensive geriatric assessments in older adults with head and neck and lung cancer to find patients with pretreatment dysfunction continued to decline through radiation treatment, lacked the ability to recover quality of life domains, and reported higher severity of symptoms [35]. Neve et al. demonstrated worse postoperative outcomes and lower radiation completion rates in older adults with abnormal baseline G8 screening scores [36]. Both studies demonstrated that an abbreviated GA tool can have predictive capacity for specific cancer-related outcomes. Future research should be focused on expanding the knowledge in this growing population of older cancer adults with specific programs to support these patients throughout their radiation treatment.

Despite known evidence showing increased toxicity in older patients with head and neck cancer getting multi-modality treatments, little is known about how to widen the therapeutic ratio in this population [37]. While cancer incidence in general is expected to increase in the elderly, it is estimated that HNC will specifically increase by more than 60% [1]. This is attributed to the increase in HPV-positive oropharyngeal cancer burden in older men who were never age-eligible for the current HPV vaccines [38,39]. Promising efforts in treatment de-escalation have been made in HPV-associated oropharyngeal cancer, which may prove to benefit the elderly [40]. However, older patients frequently are not included in large prospective randomized trials and limit the ability to extrapolate outcomes to older patients. A meta-analysis of 93 clinical trials in HNC showed only 4% of patients enrolled were ≥ 70 years of age [41]. Studies evaluating surgery, radiation, and CRT treatment modalities appear to be equally efficacious in older and younger patients, and argue that perceptions about treatment tolerance should not be made solely on chronological age [37]. Comorbidities and functional age should serve as more reliable predictors of treatment tolerance and the development of toxicities.

Chemotherapy is typically given concurrently with radiotherapy in locally advanced cases of HNC. Pignon et al. demonstrated there to be an overall survival benefit of approximately 5% at 5 years, however this benefit was not evident among older adults [41]. Adults 71 years of age and older had no statistical benefit in 5-year survival rates with the addition of chemotherapy [41]. While this older population is at increased risk of noncancer related deaths, a lack of survival benefit may also be due to the limited number of evaluable patients. As evidenced by our study, older adults are less frequently given first-line cisplatin chemotherapy in fear of developing severe toxicities. While practice-oriented recommendations have been made for cisplatin-ineligible patients, formal testing is not routinely performed in clinical practice [42]. For example, a physically fit 75-year-old with expected longevity may receive a local regional and overall survival benefit from CRT, however his chronological age alone and lack of randomized data may preclude the medical oncologist from prescribing chemotherapy. To balance the risks and benefits of more effective or toxic treatment among older patients with comorbidities, we require better tools to help predict which patients will tolerate aggressive therapy. We have shown that sarcopenia can serve as a surrogate marker for potential chemotherapy toxicity and frailty, especially given the increased prevalence in older adults.

A major limitation of this study is the lack of pre-treatment geriatric assessments to help predict patients experiencing increased toxicity from treatment. However, patients included in this study were treated before major cancer society recommendations [10], and the outcomes help guide prospective measures to study. Future research should incorporate co-management with a trained geriatrician that may provide the support needed for a patient to complete an aggressive treatment plan. Additionally, our study is limited by the retrospective nature performed at a single institution. Older adults in our population had worse baseline performance status compared to younger adults, and while this may be expected, it limits conclusions drawn between groups. Body composition measurements for skeletal muscle index, skeletal muscle density, and skeletal muscle gauge have not been widely standardized, nor has serum inflammatory marker measures, which limits the ability to compare across data. Nevertheless, these markers do provide objective measures that can be tracked throughout a patient’s cancer journey and may reflect their physiologic reserve.

## 5. Conclusions

Rates of chemotherapy and radiation treatment toxicity are high in patients undergoing definitive treatment for locally advanced head and neck cancer, and older adults are particularly vulnerable. Our results would suggest needed prospective research using screening tools to identify subsets of older patients who benefit from intensive supportive care to maximize oncologic outcomes while maintaining quality of life. Additional multi-disciplinary care from a trained geriatrician may improve deficits in geriatric functional domains that may lead to less side effects from cancer treatments.

## Figures and Tables

**Table 1:** Patient population characteristics stratified by younger (<70 years old) and older age groups (≥70 years old). Bolded variables represent *p*-value < 0.05.

	Younger Patients N (%)	Older Patients N (%)	*p* Value
Total Patients	221 (100)	51 (100)	
Gender			0.52
Male	182 (82.4)	40 (78.4)	
Female	39 (17.6)	11 (21.6)	
Race			**0.035**
White	195 (88.2)	50 (98.0)	
Non-white	26 (11.8)	1 (2.0)	
Smoking Status			
Never	62 (28.1)	19 (37.3)	0.16
Former	103 (46.6)	25 (49.0)	
Current	56 (25.3)	7 (13.7)	
BMI			0.25
Non-obese (<30 kg/m^2^)	146 (66.1)	38 (74.5)	
Obese (>30 kg/m^2^)	75 (33.9)	13 (25.5)	
ECOG status			**0.022**
0–1	202 (91.4)	41 (80.4)	
2+	19 (8.6)	10 (19.6)	
TNM Stage			0.18
Stage III	53 (24.0)	8 (15.7)	
Stage IVA	153 (69.2)	42 (82.4)	
Stage IVB	15 (6.8)	1 (2.0)	
Subsite			0.48
Larynx/hypopharynx	49 (22.2)	12 (23.5)	
Oropharynx	146 (66.1)	30 (58.8)	
Other	26 (11.8)	9 (17.6)	
p16 oropharynx status			0.050
Yes	105 (47.5)	32 (62.7)	
No	116 (52.5)	19 (37.3)	

**Table 2:** Multivariate predictors of chemotherapy and radiation toxicity breaks. * Selected variable identified by random forest method. Bolded variables represent *p*-value < 0.05.

	Chemotherapy Toxicity		Any Radiation Break		Prolonged Radiation Breaks	
	Odds Ratio (95% CI)	*p*-Value	Odds Ratio (95% CI)	*p*-Value	Odds Ratio (95% CI)	*p*-Value
Age ≥ 70	**2.76 (1.36–5.60)**	**<0.01**	**2.23 (1.19–4.19)**	0.012 *	**3.21 (1.37–7.51)**	**<0.01**
ECOG Performance status				0.11		
0–1			0.50 (0.22–1.18)			
≥2			Ref			
Smoking Status		**<0.01**				
Never	**Ref**					
Former	**0.61 (0.32–1.15)**					
Current	**2.32 (1.11–4.89)**					
BMI						0.18
<30					0.47 (0.16–1.42)	
≥30					Ref	
Low SMI		<0.01 *				0.044 *
Yes	**2.14 (1.25–3.66)**				**2.70 (1.03–7.11)**	
No	**Ref**				**Ref**	
SMG				0.06		0.24
High			Ref		Ref	
Low			1.67 (0.97–2.88)		1.71 (0.70–4.19)	
Pre-treatment albumin						0.033 *
>3.5 g/dL					**Ref**	
≤3.5 g/dL					**2.83 (1.09–7.37)**	

## Data Availability

All data is stored separately in a data repository and are available from the corresponding author on reasonable request.

## Supplementary Materials

The following supporting information can be downloaded at: https://www.mdpi.com/article/10.3390/cancers14092094/s1, Table S1. Univariate predictors of chemotherapy and radiation toxicity breaks. Bolded variables represent *p*-value < 0.05.
