# Supplementary material for: Sarcopenia and Treatment Toxicity in Older Adults Undergoing Chemoradiation for Head and Neck Cancer: Identifying Factors to Predict Frailty
Source: Cancers (Basel). 2022 Apr 22;14(9):2094. doi: 10.3390/cancers14092094 (PMC9103923; doi:10.3390/cancers14092094)
Supplement: Supplementary file 1 [file cancers-14-02094-s001.zip › cancers-1677164-supplementary.pdf]

Supplementary Material

Table S1. Univariate predictors of chemotherapy and radiation toxicity breaks. Bolded variables represent p-value < 0.05.

	Chemotherapy Toxicity		Any Radiation Break		Prolonged Radiation Breaks	
	Odds Ratio (95% CI)	P-value	Odds Ratio (95% CI)	P-value	Odds Ratio (95% CI)	P-value
Age ≥70	**2.57 (1.38-4.78)**	**<0.01**	**2.39 (1.28-4.44)**	**<0.01**	**4.02 (1.86-8.73)**	**<0.01**
Gender		0.36		0.19		0.36
Male	Ref		Ref		Ref	
Female	1.33 (0.72-2.50)		1.51 (0.81-2.83)		1.50 (0.63-3.56)	
Race		0.14		0.64		0. 14
White	Ref		Ref		Ref	
Non-white	0.49 (0.19-1.25)		0.82 (0.35-1.93)		0.12 (0.01-2.01)	
ECOG Performance status		0.32		**0.04**		0.14
0-1	0.67 (0.31-1.47)		**0.44 (0.20-0.96)**		0.48 (0.18-1.28)	
≥2	Ref		**Ref**		Ref	
Stage		0.81		0.38		0.29
III	Ref		Ref		Ref	
IV	0.93 (0.52-1.68)		1.32 (0.71-2.45)		1.71 (0.63-4.65	
Subsite		0.32		0.84		0.41
Larynx/Hypopharynx	Ref		Ref		Ref	
Oropharynx	0.85 (0.47-1.53)		0.87 (0.47-1.60)		0.66 (0.28-1.55)	
Other	0.50 (0.20-1.24)		1.05 (0.44-2.48)		1.20 (0.39-3.70)	
Smoking Status		**<0.01**		0.45		0.36
Never	**Ref**		Ref		Ref	
Former	**0.67 (0.37-1.23)**		0.24 (0.68-2.27)		0.54 (0.23-1.25)	
Current	**2.24 (1.14-4.40)**		1.56 (0.78-3.13)		0.76 (0.29-1.97)	
P16-positive oropharynx		0.77		0.58		0.21
No	Ref		Ref		Ref	
Yes	0.93 (0.57-1.53)		0.87 (0.53-1.43)		0.62 (0.28-1.31)	
Induction chemotherapy		0.67		0.28		.81
Yes	Ref		Ref		Ref	
No	1.22 (0.48-3.08)		1.75 (0.63-4.81)		1.18 (0.29-4.75)	
Concurrent Cisplatin		0.55		0.70		0.35
Yes	Ref		Ref		Ref	
No	0.85 (0.49-1.47)		0.89 (0.52-1.56)		1.43 (0.67-3.08)	

Post-operative		0.49		0.27		0.34
Yes	Ref		Ref		Ref	
No	0.81 (0.44-1.47)		0.71 (0.39-1.30)		0.67 (0.29-1.54)	
BMI		0.28		0.57		0.030
<30	1.35 (0.79-2.31)		1.17 (0.68-2.0)		2.98 (1.1-8.0)	
≥30	Ref		Ref		Ref	
Low SMI		**<0.01**		0.41		**<0.01**
Yes	**2.2 (1.28-3.75)**		1.25 (0.74-2.11)		**3.72 (1.48-9.36)**	
No	**Ref**		Ref		**Ref**	
Low SMD		0.58		0.06		0.17
Yes	Ref		Ref		Ref	
No	0.86 (0.52-1.45)		0.61 (0.36-1.03)		0.60 (0.28-1.25)	
SMG		0.12		**0.01**		**0.018**
High	Ref		**Ref**		**Ref**	
Low	1.50 (0.89-2.49)		**1.94 (1.15-3.29)**		**2.59 (1.18-5.69)**	
NRL Ratio		0.06		0.23		0.36
≥ 3	Ref		Ref		Ref	
< 3	0.62 (0.37-1.03)		0.73 (0.44-1.22)		0.71 (0.33-1.50)	
Pre-treatment albumin		0.37		0.12		**0.023**
> 3.5 g/dL	Ref		Ref		**Ref**	
≤ 3.5 g/dL	1.40 (0.66-2.95)		1.81 (0.86-3.81)		**2.84 (1.15-6.99)**	
